# Chronic snus use in healthy males alters endothelial function and increases arterial stiffness

**DOI:** 10.1371/journal.pone.0268746

**Published:** 2022-06-03

**Authors:** Lukasz Antoniewicz, Mikael Kabele, Ulf Nilsson, Jamshid Pourazar, Gregory Rankin, Jenny A. Bosson, Magnus Lundbäck

**Affiliations:** 1 Division of Internal Medicine, Department of Clinical Sciences, Karolinska Institutet, Danderyd University Hospital, Stockholm, Sweden; 2 Division of Pulmonology, Department of Medicine II, Medical University of Vienna, Vienna, Austria; 3 Division of Medicine, Department of Public Health and Clinical Medicine, Umeå University, Umeå, Sweden; 4 Division of Cardiovascular Medicine, Department of Clinical Sciences, Karolinska Institutet, Danderyd University Hospital, Stockholm, Sweden; Medical University Innsbruck, AUSTRIA

## Abstract

**Background:**

Snus usage is commonly touted as a safer alternative to cigarette smoking. However, recent studies have demonstrated possible adverse cardiovascular effects in chronic snus users. The present study evaluates the effects of chronic snus use on vascular function by assessing central arterial stiffness and endothelial vasodilatory function in healthy chronic snus users as compared to matched non-users.

**Methods and results:**

Fifty healthy males (24 snus users, 26 age-matched controls) with a mean age of 44 years were included in the study. Arterial stiffness was assessed employing both pulse wave velocity and pulse wave analysis. Endothelial vasodilatory function was measured by venous occlusion plethysmography, utilizing intra-arterial administration of acetylcholine, glyceryl trinitrate and bradykinin to further gauge endothelium-dependent and -independent vasodilatory function. Arterial stiffness was significantly higher in chronic snus users as compared to controls: pulse wave velocity [m/s]: 6.6±0.8 vs 7.1±0.9 resp. (*p* = 0.026), augmentation index corrected for heart rate [%]: 0.1±13.2 vs 7.3±7.8 resp. (*p* = 0.023). Endothelial independent vasodilation, i.e. the reaction to glyceryl trinitrate, was significantly lower in snus users as measured by venous occlusion plethysmography.

**Conclusions:**

The results of this study show an increased arterial stiffness and an underlying endothelial dysfunction in daily snus users as compared to matched non-tobacco controls. These findings indicate that long-term use of snus may alter the function of the endothelium and therefore reinforces the assertion that chronic snus use is correlated to an increased risk of development of cardiovascular disease.

## Introduction

Cigarette smoking causes approximately 6 million deaths each year worldwide [1]. These deaths are mainly attributed to heart and lung disease as well as various forms of cancer [2]. The clear association between cigarette smoking and impaired health has for some time now been an unquestioned fact. The last decades of increasing regulations and improved public awareness have caused a dramatic decline in cigarette sales. In response to this, large transnational tobacco companies have been searching for alternative means of marketing and merchandising their product. One such strategy is to advance the global market of Swedish moist snuff, a tobacco product placed under the lip, most commonly referred to as snus [3].

In Sweden, the use of snus is widely spread and can be traced back to the beginning of the 18^th^ century. Currently, approximately 22% of Swedish men and more than 4% of Swedish women use snus on a daily basis. This regular usage of snus has been steadily increasing since 2010 in both sexes [4]. Although the sale of snus is prohibited within the European Union (EU), Sweden did negotiate an exemption to this rule upon joining in 1995. As a result, snus is mainly manufactured in Sweden as well as in Norway, which is not an EU member. Despite persistent and intense lobbying efforts by the tobacco industry to challenge this ban, the EU Advocate General has opted to uphold this legislation [3]. Therefore, until recently, this product has almost exclusively been found in the Scandinavian countries.

Snus was first introduced to the US market in 2006, initially only available in a handful of cities used as test markets. Since then, snus has been heavily marketed by tobacco companies towards several different potential customer groups including women, young adults as well chewing tobacco users [5]. As the tobacco industry looks to establish these products in new markets, it has been greatly debated whether snus is a safer alternative to cigarette smoking [6].

At present, several studies have demonstrated a link between snus use and increased risk of type 2 diabetes, heart failure as well as an increased mortality following myocardial infarction (MI) and stroke [7–11]. However, there are also studies that question these associations and which tend to be dismissive of the overall risk for cardiovascular disease (CVD) [12, 13]. Mechanistic studies investigating possible pathophysiological effects following chronic snus use are scarce. Thus far, the acute biological effects found immediately following snus use are increased blood pressure and heart rate as well as endothelial dysfunction demonstrated by flow mediated dilation [14, 15]. Furthermore, daily snus users exhibit a chronically altered flow mediated dilation compared to non-users [16, 17]. These findings indicate an intrinsic association between chronic snus use and endothelial dysfunction.

Increased arterial stiffness is an independent risk factor for the development of CVD [18]. Determining central arterial stiffness by pulse wave velocity (PWV) and pulse wave analysis (PWA) is a well-established and non-invasive method. Another method for assessing vascular function is measuring forearm blood flow (FBF) with venous occlusion plethysmography, which is generally accepted as the “gold standard” for the evaluation of endothelial function [19]. Through intra-arterial administration of locally active vasodilatory drugs, venous occlusion plethysmography is used to assess endothelium-dependent and independent vasodilation in the forearm. This comprehensive technique allows for analysis of different pathways and aspects of endothelial dysfunction as well as evaluating general vasomotor function. Venous occlusion plethysmography has previously been employed to demonstrate endothelial damage in e.g. cigarette smokers and individuals exposed to combustion emission air pollution [20, 21]. To our current knowledge, no study has been performed in chronic snus users utilizing these methods to assess central arterial stiffness or vascular function.

This study aims at investigating the effects of long-time habitual snus use with several well-established methods for measuring vascular health.

## Materials and methods

### Study design

Sample size analysis was performed using G*Power 3.1 for a two-tailed ANOVA with repeated measures and between factor analysis [22]. A comparable study, analyzing forearm blood flow with venous occlusion plethysmography in smokers and non-smokers was used for effect size estimation [23]. A priori calculation using an effect size f of 0.42 showed that a total sample size of 50 is needed with a statistical power of 95% at a p<0.05 significance level.

Twenty-four healthy male chronic snus users (≥15 years of snus use) and 26 age-matched healthy controls between the age of 30 to 65 years were included in the study. Study exclusion criteria included prior smoking > 1year, hypertension, any form of cardiovascular, metabolic or respiratory disease, BMI >30 as well as active allergy or inflammation within four weeks prior to the study. Upon enrolment, study participants had to complete a health wellness form and were investigated with ECG, dynamic spirometry, blood pressure control and blood tests (total blood count, white blood count, Na, K, creatinine, apolipoprotein A and B, HbA1C, INR, aPTT). Length, weight and waist circumference were checked upon enrolment. Self-reported tobacco-use as well as alcohol consumption and level of physical activity was recorded. Prior to measurements, study participants had to abstain from all forms of nicotine, alcohol and caffeine for 24 hours and from vigorous physical activity for 48 hours. All measurements were performed in a quiet, temperature-controlled room with volunteers resting comfortably in a semi-supine position.

### Arterial stiffness

Following 20 minutes of rest, blood pressure was measured using a semi-automated non-invasive oscillometric sphygmomanometer (Boso-Medicus, Boso, Jungingen, Germany) after which arterial stiffness measurements commenced.

In accordance with the manufacturer’s instructions, pulse wave analysis was assessed at the right radial artery with applanation tonometry using a transcutaneous micromanometer (Millar Instruments, Texas, USA) employing the SphygmoCor™ system (AtCor Medical, Sydney, Australia). Briefly, pulse wave analysis obtains the radial artery pulse wave and systematically converts this to an aortic pulse pressure waveform through a validated mathematical transfer function. The waveform is composed of a forward pressure wave, originating from the ventricular contraction, and a reflected wave caused by the peripheral vascular resistance. Augmentation index (AIx), expressed as a percentage, relates how much of the pulse pressure rise is attributed to the reflected wave. As Augmentation index is inversely proportional to heart rate (HR), it is commonly normalized for a heart rate at 75bpm (AIx@75). Measurements were accepted according to the SphygmoCor^™^ quality control criteria.

Pulse wave velocity (PWV) was measured using the Vicorder^™^ system (Skidmore Medical, Bristol, UK). This well-validated, non-invasive method obtains the pulse at sensor points on two inflatable cuffs, one placed around the neck over the carotid artery and the other at the upper thigh registering the femoral artery. As the distance between these two points is measured and recorded, PWV can then be calculated to reflect the measure of the pulse pressure wave’s rate of travel.

Three independent measurements of PWA, PWV and blood pressure were obtained from each volunteer and reported as mean values, as is standard practice.

### Forearm blood flow

All participants underwent cannulation of the brachial artery using a 27-standard wire gauge needle. Following 30 minutes of saline infusion, acetylcholine at 5, 10 and 20 μg/min; glyceryl trinitrate (GTN) at 4, 8 and 16 μg/min and bradykinin at 100, 300 and 1000 pmol/min were infused for 6 minutes at each dose. All infused vasodilators were separated by 20 minutes of saline infusion and given in a randomized order. FBF was assessed in both arms (infused and non-infused) by venous occlusion plethysmography using a mercury-in-silicone gauge as previously described [19].

### Statistical analysis

Statistical calculations were performed with SPSS Statistics (24.0, IBM Corporation, NY, US) and GraphPad Prism (8.0, GraphPad Software Inc., CA, US) software. Data was checked for normality applying Shapiro-Wilk test. Skewed variables were checked for outliers and were analyzed by means of a non-parametric test (Mann-Whitney U Test) and normally distributed variables were compared with independent samples T-test. Two-way ANOVA for repeated measures was performed on measurements of FBF. If Mauchly’s test for sphericity was violated, Greenhouse-Geisser corrected results were presented. Skewed variables in multiple measures ANOVA were analyzed following logarithmic transformation. Multiple regression analysis (Method: Stepwise Enter) was applied for arterial stiffness measurements. Prior to analysis independent variables were checked for collinearity. p-values of <0.05 were considered to be statistically significant. Blinded investigators performed all analyses.

### Ethics statement

The study was approved by the local Ethics Review Board in Umeå and performed in accordance to the Declaration of Helsinki and with the written, informed consent of all participants. The whole study was performed at Umeå University and at Umeå University Hospital, Sweden.

## Results

Baseline characteristics are presented in Table 1. The loose type of snus was used by 13, and the portion form by 11 snus users (54% vs 56% resp.). In order to negate single dose size differences, snus use was classified as cans per week. The only significant factor among baseline characteristics between groups was that snus users had a significantly higher alcohol consumption compared to controls.

**Table 1:** Baseline characteristics.

	Snus users n = 24	Controls n = 26	*p*-values
Age [years]	44.8±8.5	43.4±8.6	n.s.
BMI [kg/m^2^]	25.5±2.4	25±3.3	n.s.
Waist circumference [cm]	91.8±7.7	90.3±8.6	n.s.
Alcohol consumption [ml/week]	62.3±57.3	29.3±30.3	0.017
Vigorous physical activity [h/week]	2.4±1.5	2.5±1.7	n.s.
Hemoglobin [g/L]	152.8±7.5	147.3±10.3	n.s.
Leukocyte count [x10^9^/L]	5.7±1.2	5.4±1.1	n.s.
Platelet count [x10^9^/L]	225±29.4	219.3±45.7	n.s.
Creatinine [μmol/L]	85.8±10.2	87.7±10	n.s.
Apolipoprotein A [g/L]	1.5±0.2	1.5±0.2	n.s.
Apolipoprotein B [g/L]	1.1±0.3	0.9±0.2	n.s.
Apolipoprotein B/A ratio	0.7±0.2	0.6±0.2	n.s.
HbA1c [mmol/mol]	35.6±3.7	36.2±2.6	n.s.
Snus use [years]	29.3±8.5	0±0	n.s.
Snus use [cans/week]	5.8±2.4	0±0	n.s.

Mean values ± SD.

### Arterial stiffness

**Fig 1:**
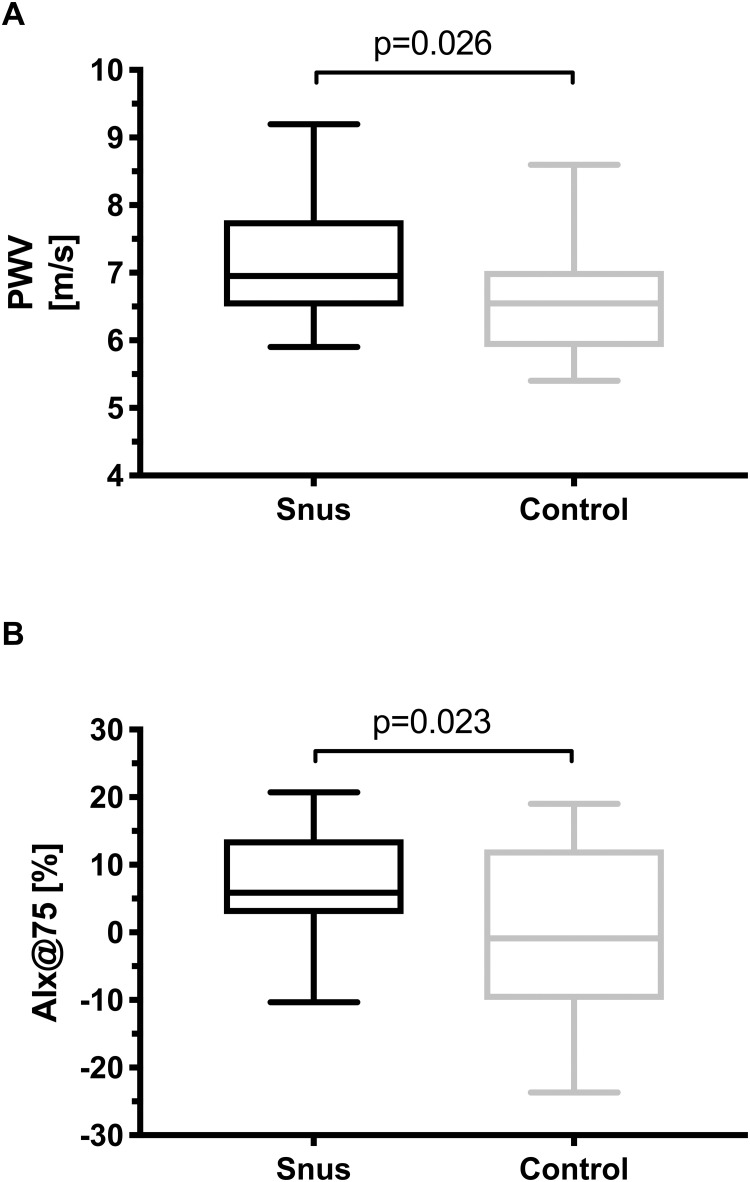
Arterial stiffness in chronic snus users and controls. Boxplots for (A) pulse wave velocity (PWV) and (B) heart rate corrected augmentation index (Aix@75). Whiskers represent minimum to maximum values.

Snus users had significantly higher pulse wave velocity (PWV) and augmentation index corrected for heart rate (AIx@75) compared to controls (Fig 1). There was no significant difference in systolic and diastolic blood pressure or heart rate between snus users and controls (SBP [mmHg]: 123.9 vs 123.7, DBP [mmHg]: 78.5 vs 76.1, HR [bpm]: 54.9 vs 55.5 resp.).

Waist circumference was significantly correlated with BMI, apolipoprotein ratio and vigorous physical activity (S1 Table). Age, apolipoprotein ratio, vigorous physical activity and alcohol consumption were significantly correlated with HbA1c. Snus use, age, alcohol consumption and waist circumference were used in multiple regression analysis to predict PWV and AIx. Age and snus consumption were the only independent variables that significantly predicted arterial stiffness:

$$\text{PWV}:5.378+\left(\text{age}\;\text{x}\;0.028\right)+\left(\text{snus}\;\text{use}:0.491\right);\text{F}\left(2,47\right)=4.968,\text{p}=0.011,{\text{R}}^{2}=0.175.$$

$$\text{AIx}@75:-29.47+\left(\text{age}\;\text{x}\;0.681\right)+\left(\text{snus}\;\text{use}:6.244\right):\text{F}\left(2,47\right)=12.888,\text{p}<0.001,{\text{R}}^{2}=0.354$$

### Forearm blood flow

**Fig 2:**
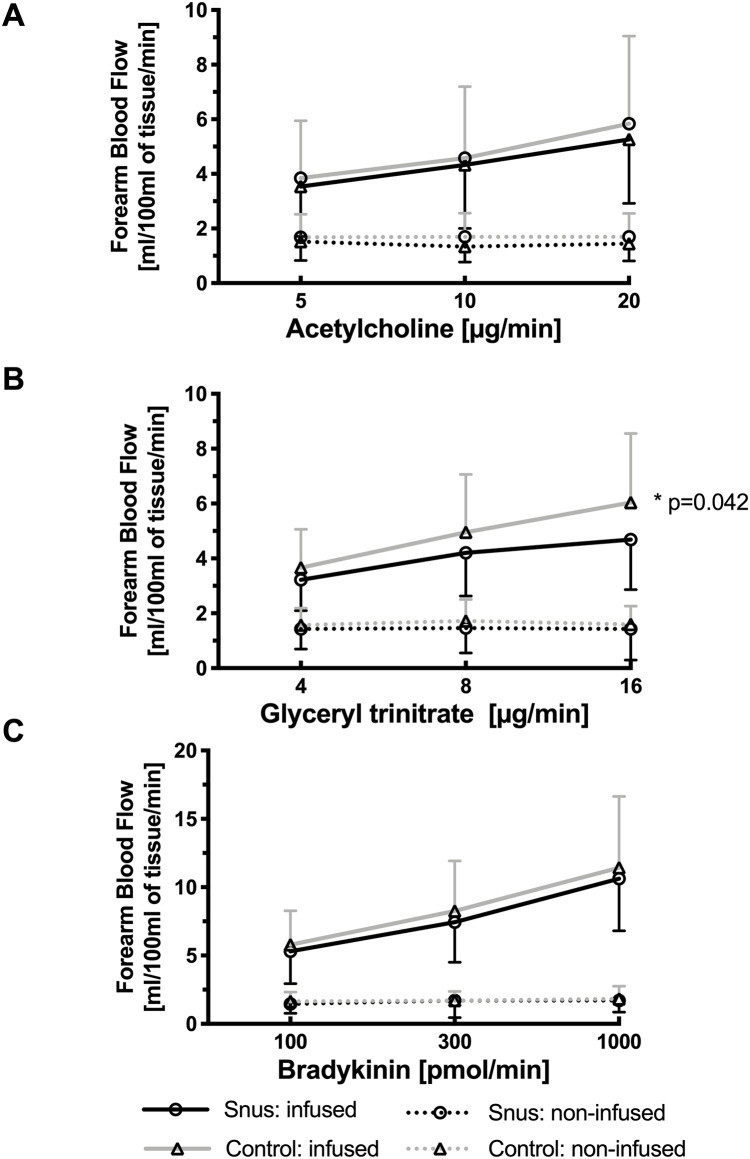
Vasodilatory function in chronic snus users and controls. Forearm blood flow (mean values ± SEM) in snus users and controls during unilateral, intra-brachial infusion of vasoactive drugs in infused (solid line) and non-infused (dotted line) arms. Significant *p*-values are given for students T-test applied at each vasoactive drug dose.

All vasoactive drugs caused a dose-dependent increase in FBF (p<0.001) in repeated measures ANOVA. However, at the highest dose of GTN (16μg/ml) snus users had significantly lower FBF compared to controls (Fig 2).

## Discussion

The present study is the first to show elevated arterial stiffness (PWV and AIx@75) in normotensive chronic snus users as compared to age-matched healthy controls. Chronic snus users also exhibited an impaired response to the endothelium-independent vasodilator GTN in FBF measured by venous occlusion plethysmography.

This study casts new light on the continuing debate whether snus use is a modifiable risk factor for CVD, particularly in regard to myocardial infarction (MI) and stroke. Thus far, cohort studies of snus users indicate no overall increase in the risk for onset of CVD compared to non-users. However, mortality rates do appear elevated in long term snus users that in fact go on to develop MI or stroke [7, 11–13]. This was further supported by Arefalk et al. who monitored snus users that gave up the habit compared to those who did not quit following an MI. They observed a nearly 50% reduction of mortality rates in the cessation group as compared to those that continued to use snus [10]. They also determined that the risk reduction was of the same magnitude as seen following smoking cessation post-MI. In the current study, we demonstrate that long term snus use is associated with increased arterial stiffness and impaired endothelial function, which strengthens the argument that snus use does indeed need to be considered a risk factor for CVD. The results also offer a pathophysiological explanation as to how snus use may lead to an elevated risk of death following MI or stroke.

Increased arterial stiffness is a blood pressure independent risk factor for the onset of CVD [18]. This stiffening of the arteries inversely impacts both systolic and diastolic pressure thus steadily leading to an increased workload, heightened oxygen demand of the left ventricle as well as diminishing coronary flow during diastole [24]. It is well-established that cigarette smoking leads to increased arterial stiffness as measured by PWV and AIx@75, yet the effects of snus use on arterial stiffness have not been as thoroughly investigated [25]. Regression analysis of our data demonstrates that age as well as snus use can predict an increase in arterial stiffness. Arterial stiffness has been shown to augment with age due to the physiological loss of elastic properties in the vascular tissue due to years of repeated stretching [26]. In this study we find that snus usage is comparable to an increase of Aix@75 throughout the time of 9.2 life years and 17.5 life years for PWV.

The monolayer of endothelial cells is key for sustaining systemic as well as local homeostasis. Endothelial dysfunction can affect both the macrovasculature as well as the microvasculature and has been associated with nearly every known risk factor for CVD, making it an independent and valuable predictor of cardiovascular events [27]. There are two previous studies that have shown impaired endothelial function in chronic snus users as measured by flow mediated dilation [16, 17]. However, this is currently the first study to investigate vasomotor function in chronic snus users by means of forearm venous occlusion plethysmography, recognized as the ‘gold standard’ method for the assessment of endothelial function [19]. We demonstrate that chronic snus users have attenuated FBF following GTN infusion as compared to the control group, yet no significant differences were found between the two groups following infusions of acetylcholine and bradykinin.

Nitric oxide (NO) is a signaling molecule released from the endothelium by the enzyme nitric oxide synthase, which generates smooth muscle relaxation and acts as a local anti-inflammatory agent [27]. Reduced bioavailability of NO is considered to be the hallmark feature of endothelial dysfunction. Acetylcholine and bradykinin are both endothelium-dependent dilators (EDD), which denotes a dependence on the function of nitric oxide synthase in the endothelium. In contrast, GTN is an endothelium-independent dilator (EID) and acts as a NO donor, and therefore is a test of NO sensitivity further along the pathway.

Our novel finding that chronic snus use is associated with impaired vasodilation following GTN infusion may offer a further explanation for the increased mortality rates seen in snus users during acute MIs as well as post-MI [7, 10]. GTN, also known as nitroglycerin, is a commonly used medication both in hospitals and as needed in daily life to prevent and treat chest pain caused by an inadequate supply of oxygen to the heart muscles, such as in angina pectoris and acute MI. It is well known that habitual, repeated usage of GTN causes nitrate tolerance and attenuated EID [28]. One hypothesis to the underlying mechanism of this is that an important enzyme in the signaling cascade for vasodilation, the cGMP-dependent protein kinase (PKG), may be diminished upon repeated GTN exposure [29]. It has been shown in rats that nicotine may also alter EID through this pathway, as chronic nicotine altered aortic muscle cell relaxation through the PKG pathway [30]. This is also supported by Halimi et al. who found that nicotine may alter the PKG pathway by demonstrating decreased urinary excretion of cGMP after nicotine administration in non-smoking human subjects [31]. Therefore, it is possible that chronic nicotine exposure may have similar effects as recurrent GTN use on the PKG pathway resulting in a diminished vasodilation response to GTN in chronic snus users.

Lind et al. studied FBF in cigarette smokers compared to matched non-smokers, they showed an inverse correlation of the duration of smoking to impairment of EDD, with a trend seen towards reduced EID [20]. It is possible that combustion-derived compounds found in cigarette smoke are responsible for these effects on the EDD [32]. Nicotine, a key component in snus, is known to prompt a systemic sympathomimetic reaction and may induce increased arterial stiffness and endothelial dysfunction [33]. A study where rats were chronically exposed to nicotine-free cigarette smoke extract showed impaired EDD, but not EID [34]. Furthermore, due to prolonged absorption, additives that raise the product pH as well as sustained plasma levels of nicotine and its metabolites, snus users are exposed to higher nicotine levels as compared to cigarette smokers [35, 36]. Thus, the adverse effects of snus use on EID may be markedly attributable to the high circulating levels of nicotine.

Clinical experimental studies examining vascular function following exposure to nicotine or nicotine replacement therapy are scarce. Though it has been shown that the intake of a single 2mg nicotine tablet caused an acute elevation in arterial stiffness in healthy volunteers as opposed to placebo [37]. This could suggest that nicotine itself can also alter arterial stiffness, which is further supported by findings from in vitro and animal studies. Nicotine exposure in vitro has been demonstrated to decrease the elastic properties of smooth muscle cells and increase proliferation of endothelial cells [38, 39]. Furthermore, rats exposed to repeated intravenous nicotine infusions displayed aortic remodeling, a process associated with the development of hypertension [40].

Baseline characteristics in this study showed that chronic snus use was significantly associated with a higher alcohol use than in non-snus users. This finding may not be so unexpected, as tobacco use has been linked with increased alcohol consumption [41]. More importantly, the overall alcohol consumption was not at risk-behavior levels and was not correlated with arterial stiffness in regression analysis.

### Study limitations

The present study investigated male subjects. Endothelial function has been demonstrated to be affected by sex hormones, several of these differences may explain the divergence of CVD presentation in women relative to men. Therefore, it is pertinent to perform these as separate studies, so as to be able to power each group in order to correctly assess the effects on vasomotor function. As snus usage among women has shown an increasing trend during the last decade, it is undoubtedly important to continue with similar studies performed in females.

We did not evaluate additional factor that furthermore may influence endothelial function: level of education, monthly income and diet [42, 43]. Instead, waist circumference, BMI and self-reported levels of physical activity were assessed which partly correlate with these factors [44, 45].

## Conclusions

The present study demonstrates an impaired endothelial function as well as an increased arterial stiffness in chronic snus users as compared to matched non-tobacco controls. Both findings designate early risk factors for CVD. Furthermore, chronic snus usage significantly impacted endothelial independent dilation, resulting in a decreased vasodilatory response to GTN. This study not only aligns with the epidemiological data suggesting that chronic snus use poses a risk factor for mortality related to CVD but also demonstrates a pathophysiological explanation for the deleterious impacts on the endothelium.

## Supporting information

S1 Table Spearman correlation of baseline characteristics. CC = Correlation Coefficient. Sig. = 2-tailed significance. * Correlation is significant at the 0.05 level. ** Correlation is significant at the 0.01 level.
(DOCX)
