# Supplementary material for: Chronic snus use in healthy males alters endothelial function and increases arterial stiffness
Source: PLoS One. 2022 Jun 3;17(6):e0268746. doi: 10.1371/journal.pone.0268746 (PMC9165771; doi:10.1371/journal.pone.0268746)
Supplement: S1 Table — CC = Correlation Coefficient. Sig. = 2-tailed significance. * Correlation is significant at the 0.05 level. ** Correlation is significant at the 0.01 level. (DOCX) [file pone.0268746.s001.docx]

|  | | age [years] | BMI [kg/m2] | waist circumference [cm] | apolipo B/A ratio | HbA1c [mmol/mol] | vigorous physical activity [h/week] | alcohol consumption [ml/week] |
| --- | --- | --- | --- | --- | --- | --- | --- | --- |
| age  [years] | CC | 1.000 | 0.171 | 0.248 | 0.239 | .412^**^ | -0.238 | 0.052 |
|  | Sig. |  | 0.236 | 0.083 | 0.095 | 0.003 | 0.096 | 0.719 |
| BMI  [kg/m^2^] | CC | 0.171 | 1.000 | .887^**^ | .341^*^ | 0.030 | -0.240 | 0.168 |
|  | Sig. | 0.236 |  | 0.000 | 0.015 | 0.834 | 0.093 | 0.243 |
| waist circumference [cm] | CC | 0.248 | .887^**^ | 1.000 | .429^**^ | 0.130 | -.331^*^ | 0.085 |
|  | Sig. | 0.083 | 0.000 |  | 0.002 | 0.368 | 0.019 | 0.556 |
| apolipo B/A ratio | CC | 0.239 | .341^*^ | .429^**^ | 1.000 | .344^*^ | -0.206 | 0.102 |
|  | Sig. | 0.095 | 0.015 | 0.002 |  | 0.014 | 0.152 | 0.480 |
| HbA1c  [mmol/mol] | CC | .412^**^ | 0.030 | 0.130 | .344^*^ | 1.000 | -.328^*^ | -.334^*^ |
|  | Sig. | 0.003 | 0.834 | 0.368 | 0.014 |  | 0.020 | 0.018 |
| vigorous physical activity [h/week] | CC | -0.238 | -0.240 | -.331^*^ | -0.206 | -.328^*^ | 1.000 | 0.105 |
|  | Sig. | 0.096 | 0.093 | 0.019 | 0.152 | 0.020 |  | 0.467 |
| alcohol consumption [ml/week] | CC | 0.052 | 0.168 | 0.085 | 0.102 | -.334^*^ | 0.105 | 1.000 |
|  | Sig. | 0.719 | 0.243 | 0.556 | 0.480 | 0.018 | 0.467 |  |
